# Structure and Neuroprotective Effect of Polysaccharide from Viscera Autolysates of Squid *Ommastrephes bartrami*

**DOI:** 10.3390/md17030188

**Published:** 2019-03-22

**Authors:** Peng Ye, Peipei Li, Wenge Yang, Yue Zhao, Yuqin Zhao, Kunlai Sun, Bin Wang, Yin Chen

**Affiliations:** 1 College of Food and Pharmacy, Zhejiang Ocean University, Zhoushan 316000, China; 17342092831@163.com (P.Y.); zy763115530@163.com (Y.Z.); zhaoy@zjou.edu.cn (Y.Z.); sunqinlai@126.com (K.S.); wangbin@zjou.edu.cn (B.W.); 2 Marine School, Ningbo University, Ningbo 315000, China; liwanzhao999@163.com (P.L.); yangwenge@nbu.edu.cn (W.Y.); 3 Zhejiang Mariculture Research Institute, Zhoushan 316000, China

**Keywords:** polysaccharide, squid autolysate, structure, neuroprotective

## Abstract

To explore bioactive polysaccharides from the byproducts of squid processing, a heteropolysaccharide, named SV2-1, was isolated from the viscera of squid *Ommastrephes bartrami* by autolysis, anion-exchange and gel-permeation chromatography and measured for its neuroprotective activity. It was a homogeneous polysaccharide with a molecular weight of 2.3 kDa by HPSEC analysis. SV2-1 contained glucuronic acid, galactosamine and fucose in the ratio of 1.0:1.1:1.2. Its structural characteristics were elucidated by methylation analysis, gas chromatography-mass spectrometry (GC-MS), and nuclear magnetic resonance (NMR). The backbone of SV2-1 was composed of alternant →4)-α-l-Fuc*p*-(1→ and →3)-β-d-GlcUA-(1→ Most of →4)-α-l-Fuc*p*-(1→ (90%) was substituted by single α-d-GlcNAc as the branches. SV2-1 can protect against the death of PC12 induced by 6-OHDA, and effectively improves cell viability and reduces extracellular LDH release in PC12 cells after injury. Moreover, SV2-1 significantly increases SOD activity but decreases MDA levels.

## 1. Introduction

Squid is considered to be important seafood, which is well known for its wide varieties, wide spreading distribution, abundance, and fishing production. Meanwhile, it is representative of the increased waste production in the seafood industry [1,2]. The resulting byproducts largely consist of the head, fin, wings, and viscera, which contain abundant natural proteins, lipids, and minerals. The marine bio-processing industry offers great potential to utilize these byproducts. Proteolysis is en efficient way to produce bioactive peptides and free amino acids from the discarded byproducts with high nutritive value and functional properties, which can be used in the food and medicine industries [3]. Aquatic invertebrates and vertebrates’ viscera are rich sources of various enzymes such as pepsin, trypsin, chymotrypsin, collagenase and elastase. Thus, autolysis using the complex mixture of endogenous proteases under proper hydrolysis conditions can produce squid byproduct hydrolysates. Peptides with angiotensin I-converting enzyme inhibitors activity were produced from the autolysates of squid liver and mantle muscle [4]. Squid viscera autolysates also showed strong in vitro antioxidative activities [5].

Beside peptides, polysaccharides are the interesting bioactive components of the byproduct. The exploitation and research on polysaccharides from the discarded byproducts can achieve more comprehensive and full utilization of marine resource, make waste profitable, and has a good prospect of development. A new heteropolysaccharide, isolated from the ink of cuttlefish, *Sepiella maindroni*, by enzymolysis contained glucuronic acid, mannose, *N*-acetylgalactosamine, and fucose, which presented strong antimutagenic activity [6]. A new non-sulphated acidic polysaccharide with molecular weight of 55 kDa was isolated from squid pen case after enzymolysis and β-elimination. This polysaccharide contained mainly l-iduronic acid, d-glucuronic acid, d-galactosamine, d-glucosamine and significant amounts of neutral sugars such as glucose, galactose and fucose [7]. β-chitosans, which had high water- and fat-binding capacity, antioxidant and antibacterial activity and potential biomedical applications were also isolated from the squid pen [8]. Thus, squid by-products contain abundant polysaccharides.

Squid viscera, which are one of the predominant by-products and account for more than 20% of the whole body weight, are generated in squid processing. Therefore, it is essential to develop efficient methods to convert squid viscera into more profitable and marketable products [5,9]. In this paper, the polysaccharides in the squid viscera were studied.

## 2. Results

### 2.1. Extraction, Purification and Composition of the Polysaccharide

**Figure 1:**
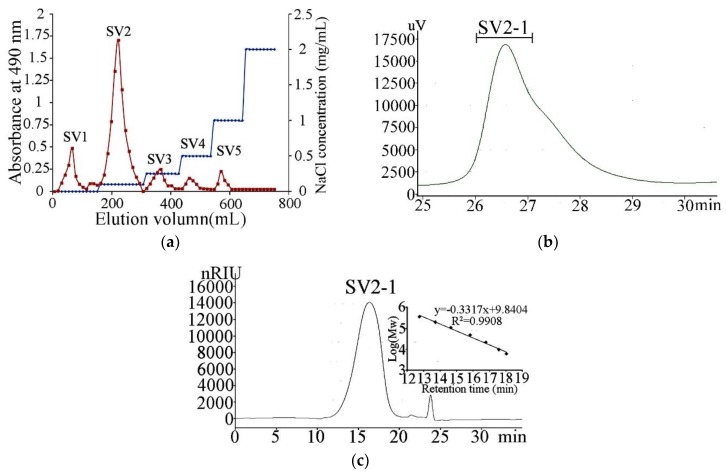
Purification and high-performance gel permeation chromatography (HPGPC) chromatogram of the polysaccharides extracted from the viscera of squid. (**a**) The crude polysaccharides were applied to a Q Sepharose Fast Flow column gradient elution; (**b**) Purification of SV2 on a Superdex75 column; (**c**) HPGPC chromatograms of SV2-1 on TSKgel G3000PWxl column (7.8 mm × 30.0 cm) and standard curve of molecular weights.

Polysaccharides are the interesting bioactive components of the by-products of cephalopod tissues like dirt, bones, skin and ink. Squid viscera were full of protein and oil. Many methods including autolysis were investigated to use these nutrient contents to enhance their commercial value. But the polysaccharides were rarely studied [10]. In this study, the polysaccharides were prepared and gathered by autolysis, the yields of which were 4.2% after removing the autolysates. The crude polysaccharides were firstly fractionated by anion-exchange chromatography using a Q Sepharose Fast Flow column coupled to an AKTA FPLC system (GE Life Sciences, Pittsburgh, PA, USA), eluted with linear and step-wise gradient (Figure 1a) of NaCl. The fractions were assayed for carbohydrate content by the phenol sulfate method. The major polysaccharides-containing fraction eluted by 0.25 mol/L NaCl which accounted for 65% of the total carbohydrate was pooled, desalted by dialysis (MWCO 3500), freeze-dried and named as SV2. SV2 was further purified on a Superdex 75 column. A major polysaccharide fraction, designated SV2-1, was obtained (Figure 1b). SV2-1 showed a single symmetrical peak on high-performance gel permeation chromatography (HPGPC) (Figure 1c). Based on the calibration curve of dextran standards, the molecular weight was calculated to be about 23 kDa. Analysis of monosaccharide composition revealed SV2-1 was composed of glucuronic acid, galactosamine and fucose in the ratio of 1.0:1.1:1.2. The monosaccharide composition revealed SV2-1 was a characteristic polysaccharide consisting of an acidic sugar, an alkaline sugar and a neutral sugar at almost the same ratio which was similar to the fucosylated chondroitin sulfates in sea cucumbers [11]. Many heteropolysaccharides from marine cephalopods also had similar monosaccharide composition. Uronic acid and amino sugar were common in these polysaccharides. A new heteropolysaccharide, named SIP, was isolated from the ink of cuttlefish, *Sepiella maindroni* was composed of glucuronic acid, mannose, *N*-acetylgalactosamine, and fucose [6]. Polysaccharide from *Octopus ocellatus* was composed of glucuronic acid, *N*-acetylglucosamine, fucose and minor galactose. Minor amounts of protein (5%) but no sulfation group was detected in SV2-1 [12].

### 2.2. Infrared (IR)

**Figure 2:**
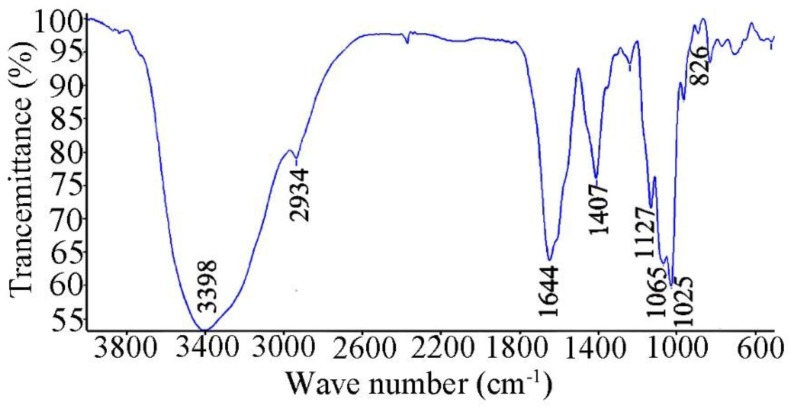
Infrared (IR) spectrum of SV2-1.

Fourier transform infrared (FTIR) spectroscopy provides important information about the conformation and functional groups of the polysaccharide. The FTIR spectrum (Figure 2) showed a strong band at 3398 cm^−1^ attributed to the hydroxyl stretching vibration of the polysaccharide. The band at 2934 cm^−1^ was due to C–H stretching vibration. The absorption at about 1644 cm^−1^ was attributed to the stretching vibration of the carbonyl bond and the bending vibration of the N–H bond, respectively. The signals at 1127, 1065 and 1025 cm^−1^ were due to the stretch vibration of C–O–C linkages and the pyranoid ring. There are two types of end carbon-glucoside bonds: α and β-styles, which can be judged by FTIR. In the FTIR spectrum of SV2-1, the C–H bond in α -style has an absorption peak at 826 cm^−1^ [6,12]. 

### 2.3. Gas Chromatography-Mass Spectrometry (GC-MS) Analysis of the Methylated Products

The fully methylated product of SV2-1 was hydrolyzed with acid, reduced and converted into alditol acetates. The analysis of the partially methylated alditol acetates by gas chromatography-mass spectrometry (GC-MS) showed the presence of 1,4,5-tri-*O*-acetyl-2,3,6-tri-*O*-methyl-l-fucitol and 1,3,4,5-tetra-*O*-acetyl-2,6-di-*O*-methyl-l-fucitol. The results indicate that →4)-l-Fuc*p*-(1→ and →3,4)-l-Fuc*p*-(1→ were present in the polysaccharide. The GC-MS of the carboxyl-reduced polysaccharide SV2-1P showed the presence of the above fucose peaks and a new peak of 1,3,5-tri-Oacetyl-2,4,6-tri-*O*-methyl-d-gluctitol at a molar ratio of 0.2:1.0:1.1. This result indicates that (1→3)-linked d-GlcpA was also present in the polysaccharide. It was hard to get the linkage information of galactosamine from GC-MS directly. Because the methylated GalN needs higher temperature to gasify than the neutral sugar [13]. The result of GC-MS is shown in Table 1.

### 2.4. Nuclear Magnetic Resonance (NMR)

**Figure 3:**
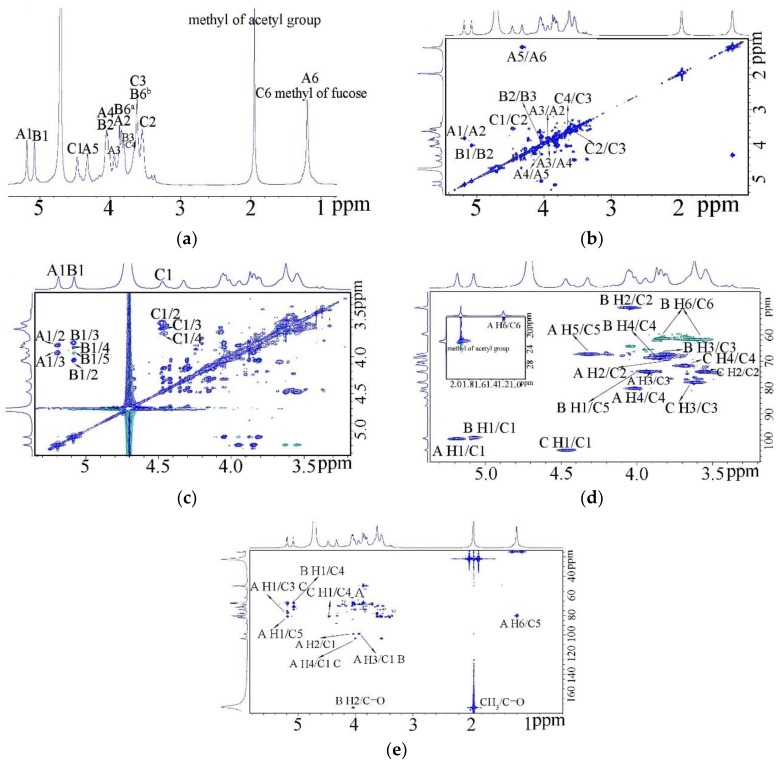
Nuclear magnetic resonance (NMR) spectra of the polysaccharide SV2-1. (**a**) ^1^H NMR spectrum; (**b**) ^1^H–^1^H COSY spectrum; (**c**) ^1^H–^1^H TOCSY spectrum; (**d**) ^1^H–^13^C HSQC spectrum; (**e**) ^1^H–^13^C HMBC spectrum;. A–C correspond to →3,4)-α-l-Fuc*p*-(1→, α-d-GlcNAc-(1→ and →3)-β-d-GlcUA-(1→ respectively.

The ^1^H nuclear magnetic resonance (NMR spectrum (600 MHz) (Figure 3a) of this polysaccharide at 25 °C showed three anomeric proton signals at 5.19, 5.08 and 4.45 ppm at a 1.1:1:0.87 molar ratio. Besides the anomeric signals, the other ring proton signals were at the region of 3.4–4.4. In addition, the ^1^H NMR spectrum showed two methyl groups at 1.19 and 1.98 ppm, which represented the C6 methyl group of fucose and CH_3_ of acetyl group respectively [14,15]. 

^1^H-^1^H COSY (Figure 3b), TOCSY (Figure 3c) and HSQC (Figure 3d) allowed the assignment of the proton signals in the sugar residue spin systems. A1 at 5.19 ppm and the CH_3_ of fucose belonged to the same spin system from the ^1^H-^1^H COSY and TOCSY, which meant A residue was fucose. The HSQC showed the three anomeric protons were related to the anomeric carbons at 99.2, 98.8 and 103.9 ppm. C-2 of B was at 50.2 ppm, which revealed it was a nitrogen-bearing carbon. So B residue was galactosamine. Based on the reference, A was α-l-Fuc, B was α-d-GlcN. The anomeric proton of C had the lowest chemical shift at 4.47 ppm which related to the anomeric carbons at 103.9 ppm. It indicated C was β-d-GlcUA. The C-3 of β-d-GlcUA changed to about 78 after substituted. Thus C represented →3)-β-d-GlcUA-(1→ linkage. For residue A, the C-3 and C-4 moved to low field to 75.1 and 80 ppm, which also indicated the linkage of →3,4)-α-l-Fuc*p*-(1→. The carbons of the two methyl groups (C-6 of l-Fucp and CH3 of an acetyl group) were at 15.9 and 22 ppm, respectively. The carboxyl groups were at 174 [7,16].

The corresponding signals from the HMBC spectrum (Figure 3e) were used to confirm the sequences of the sugar residues. The evidence that H-2 at 4.03 was related to the carboxyl group of acetyl group at 174 indicated that acetyl group was linked to the amino group of galactosamine. So B was actually *N* acetyl galactosamine. Combined these informations, the proton and carbon chemical shifts of the major residues were clear and are presented in Table 2. Cross peaks were found between H-1 of residue A with C-3 of residue C, which indicated α-l-Fuc*p* was linked to *O*-3 position of →3)-β-d-GlcUA-(1→ [17]. Similar cross peaks were found between H-4 of residue A and C-1 of residue C demonstrated (1→3)-β-d-GlcUA was linked to *O*-4 of α-l-Fuc*p*. The corresponding signal between H-1 of residue C and C-4 of residue A also confirmed this. The inter-residual coupling between H-3 of residue A and C-1 of residue C suggested α-d-GlcNAc was linked to the *O*-3 of α-l-Fuc*p*. The chemical shifts of residue B, α-d-GlcNAc had no obvious variation compared with the free d-GlcNAc, which indicated no other residue was linked to α-d-GlcNAc. Thus, α-d-GlcNAc was linked to *O*-3 of →4)-α-l-Fuc*p*-(1→ as a single terminal.

**Figure 4:**
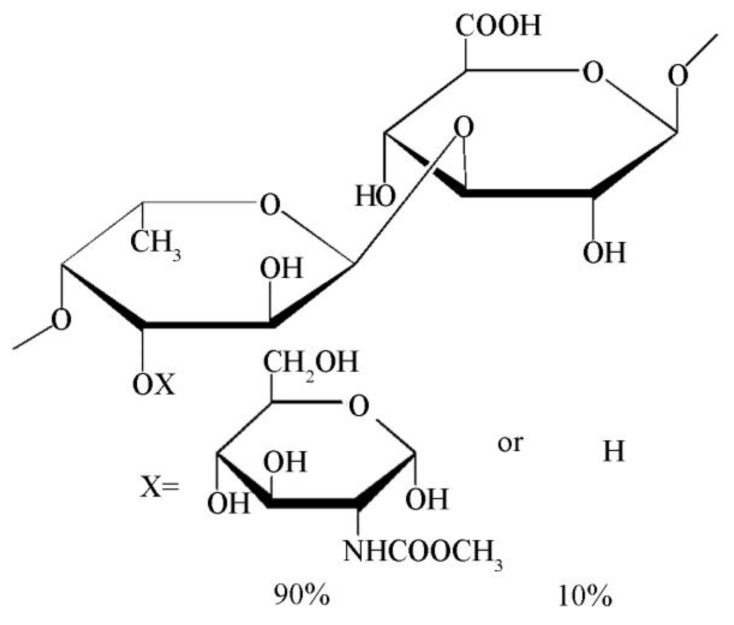
The proposed structure of SV2-1.

With the help of methylation and NMR analysis, the primary structure of SV2-1 was determinated. SV2-1 was a heteropolysaccharide with alternant →4)-α-l-Fuc*p*-(1→ and →3)-β-d-GlcUA-(1→ as the main chain. Most of →4)-α-l-Fuc*p*-(1→ (90%) was substituted by single α-d-GlcNAc as the branches. Based on these analyses, the possible structure of SV2-1 is proposed in Figure 4.

### 2.5. Neuroprotective Activity on PC12 Cell Line

The PC12 cell line as the neuronal pheochromocytoma possesses the phenotypic and physiological properties of sympathetic neurons, which was often used as a neuronal developmental model [18]. In this study, we used PC12 to evaluate the effect of the polysaccharide SV2-1 isolated from the squid viscera on preventing the injury induced by 6-hydroxydopamine (6-OHDA) [19]. 

The PC12 cells were treated with different concentrations of 6-OHDA (12.5, 25, 50, 100 and 200 μmol/L) for 24 h and with 50 μmol/L 6-OHDA for different time courses (6, 12, 24 and 48 h) as wel1. Based on the above experiment (data not shown), the best concentration (50 μmol/L) of 6-OHDA and time (24 h) were selected and the damaged model of PC12 cell line was induced accordingly. The PC12 cells were pretreated with different concentrations (0.125, 0.25, 0.5 and 1.0 mg/mL) SV2- for 1 h and then co-treated with 50 μmol/L 6-OHDA for 24 h. The viability of PC12 cells was detected by MTT assay.

**Figure 5:**
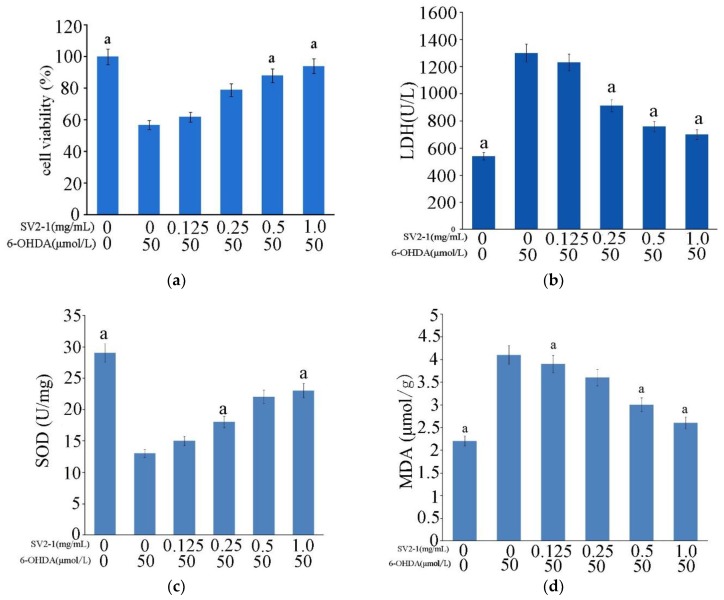
Neuroprotective effect of SV2-1 on PC12 cell line. (**a**) Effects of SV2-1 cell viability. After PC12 cells were exposed to 6-OHDA, cell viability significantly decreased; treatment with SV2-1 with different concentrations for 24 h significantly increased cell viability; (**b**) Effect of SV2-1 on the LDL level of PC12 cell. Stimulated by SV2-1, the LDH level decreased remarkably; (**c**) Effects of SV2-1 on the SOD level; (**d**) Effects of SV2-1 on the MDA level. Treatment with Sv2-1 significantly increased SOD activity and decreased MDA concentration compared with the 6-OHDA injured group. Each value is expressed as mean ± SD (*n* = 3), a means *p* < 0.05 compared with the 6-OHDA injured group.

Oxidative stress induced by 50 μmol/L 6-OHDA for 24 h significantly decreased cell viability to 55.8 ± 1.9% compared with control cells. SV2-1 (0.125–1.0 mg/mL) protected the cells against 6-OHDA-induced cytotoxicity; PC12 cells survival was improved as SV2-1 concentration increased, in which 94.7 ± 2.64% of cells were treated with 1.0 mg/mL SV2-1 (Figure 5a). These results indicated that SV2-1 displayed a protective effect against oxidative stress on PC12 cells [20].

LDH assay was performed to further investigate the protective effects of SV2-1 on oxidative injury in PC12 cells. LDH, as a marked cytoplasmic enzyme is stable in property, but will be rapidly released to the culture medium when the plasma membrane is damaged. Thus, the increased LDH activity in a culture medium corresponds to the cell necrosis to some extent [21]. After the cells were exposed to 6-OHDA injury, LDH activity increased to 1290 U/L, indicating that the cells were markedly damaged. After being treated with SV2-1, LDH level in a culture medium induced by 6-OHDA was decreased (Figure 5b). At 1.0 mg/mL, SV2-1 can reduce the LDH level to 680 U/L. These results suggested that SV2-1 effectively reduced LDH leakage induced by 6-OHDA in a concentration-dependent manner.

6-OHDA induced oxidative damage significantly decreased SOD activity to a half of the control group (Figure 5c). Oxidative abnormalities were ameliorated by SV2-1 treatment, which was manifested as obvious increase in SOD activity. MDA is widely used as a biomarker of lipid peroxidation [22]. In this study, 6-OHDA significantly increased MDA content (Figure 5d), which was significantly ameliorated by SV2-1.

## 3. Materials and Methods 

### 3.1. Preparation of Polysaccharides from the Squid Viscera Autolysates 

The frozen by product of North Pacific squid (*Ommastrephes bartrami*) were provided by Zhejiang Fudan Tourism Food Co., Ltd. Squid viscera (200 g) were thawed and mixed with distilled water at a ratio of 1:2 (w/v), and homogenized at a speed of 10,000× *g* for 1 min using a homogenizer (TM-767, Zhongshan, China). The mixture was incubated at 50 °C for 120 min to autolysis after pH was adjusted to 7.0 using 6 M NaOH. To end the autolysis, the mixtures were boiled at 100 °C for 15 min to inactivate the endogenous enzymes, and then centrifuged at 5000× *g* for 10 min to remove the insoluble substrate and upper layer of fat. The soluble squid viscera autolysates were gathered and added to Sevag solution to remove the residual protein [5]. The soluble solution was concentrated to about one-tenth of the original volume in a rotary evaporator under reduced pressure and added to ethanol (the final concentration of ethanol was 80%) to precipitate the polysaccharide. After centrifugation (6000× *g* for 15 min), the precipitate was gathered, dissolved in distilled water and centrifuged again as above. The resultant supernatant containing polysaccharide was collected, dialyzed (molecular weight cut-off 3 kDa) with distilled water for 48 h. The yellow powder of crude polysaccharide obtained by freeze-drying with a yields of 4.5% (w/w, in wet mass). 

### 3.2. Purification of Polysaccharides from the Squid Viscera Autolysates 

The crude polysaccharide (100 mg) was dissolved in distilled water (2 mL) and purified with an AKTA FPLC system using a Q Sepharose Fast Flow column (300 × 30 mm), eluted with a linear gradient of NaCl (0–2 mol/L). Fractions were collected and analyzed for carbohydrate content by the phenol–sulphuric acid method [12]. Individual peaks of the major carbohydrate fraction were pooled, dialyzed exhaustively (molecular weight cut-off 3 kDa) and further purified by high-performance liquid chromatography (HPLC)-gel permeation chromatography (GPC) on a Superdex 75 column (100 × 2 cm) eluted with 0.2 mol/L NH_4_HCO_3_ at a flow rate of 0.5 mL/min. Refractive index detection was used. The major polysaccharide fractions were gathered by automatic sampling instrument, freeze-dried and named SV2-1 [23]. Purity and molecular weight were determined by HPGPC as described [12].

### 3.3. Physicochemical Characteristics and Composition Analysis of the Polysaccharide

Complete hydrolysis of SV2-1 (5 mg) was performed with 2 mol/L trifluoroacetic acid (TFA, 1 mL) at 105 °C for 6 h. After TFA removal by repeated co-evaporation with methanol, the hydrolysate was dried under reduced pressure. The monosaccharide composition was then analyzed by HPLC through pre-column derivatization with 1-phenyl-3-methyl-5-pyrazolone using an Agilent HPLC system fitted with an Agilent XDB-C18 (4.6 × 250 mm) and Agilent XDB-UV (ultraviolet) detector. The sulfate content of the polysaccharide was determined by high-performance anion-exchange chromatography with pulsed amperometric detection (HPAEC-PAD) on a CIC-100 ion chromatograph coupled with SH-AC-1 anion exchange column (4.6 × 250 mm, 13 µm) and conductivity detector [24].

### 3.4. IR Spectroscopy Analysis

For FTIR spectroscopy, dry SV2-1 was mixed with KBr powder, grounded and then pressed into a 1 mm pellets for FTIR measurement on the Nicolet Nexus 470 instrument in the frequency range of 4000–500 cm^−1^ at the resolution of 4.0 cm^−1^ with background scanning frequency of 32. FTIR spectrum of the SV2-1 was measured by using the Nicolet Omnic software [25].

### 3.5. Carboxyl Group Reduction and Methylation Analysis

The carboxyl-group of the SV2-1 was reduced by the method of Taylor and Conrad. SV2-1 (100 mg) was dissolved in water (20 mL) and adjusted to pH 4.7 with 0.05 M HCl. After the addition of 1-ethyl-3-(3-dimethylaminopropyl) carbodiimide (300 mg), the mixture was stirred and maintained at pH 4.7 over 4 h by the slow addition of 0.05 M hydrochloric acid. Then, sodium borodeuteride (300 mg) was added to the mixture and stirred for 12 h at 20 °C. The reaction mixture was adjusted to pH 7.0 with acetic acid and dialyzed against running water for 48 h, the reduced polysaccharide (SV2-1P) was recovered by lyophilization [26]. 

The methylation analysis of SV2-1 and SV2-1P was performed according to the modified Hakomori method using sodium hydride in dimethyl sulfoxide and methyl iodide [13,27]. After methylation, the samples were hydrolyzed with TFA as described above, reduced with sodium borodeuteride and converted into partially methylated alditol acetates and analyzed by gas chromatography-mass spectrometric GC-MS on a HP6890II instrument using a DB-5ms fused silica capillary column (0.25 mm × 30 m) (Agilent Technologies Co., Ltd., USA). The temperature was increased from 100 °C to 220 °C at a rate of 5 °C /min and then maintained at 220 °C for 5 min with helium as carrier gas. The peaks on the chromatogram were identified from their retention times and the mass fragmentation patterns [28].

### 3.6. NMR Spectroscopy

Freeze-dried SV2-1(80 mg) were dissolved in 400 μL of D_2_O (99.96 atom%) and lyophilized two times to exchange exchangeable protons and transferred to a 5 mm NMR tube. ^1^H NMR, HSQC NMR, ^1^H–^1^H COSY and TOCSY were performed on a Bruker 800-MHz NMR spectrometer and acquisition of the spectra was carried out using Topspin 2.1.6 software. All spectra were acquired at a temperature of 298 K [14].

### 3.7. Neuroprotective Activity on PC12 Cell Line

Cell culture: PC12 cells (Rat adrenal pheochromocytoma cell line) were purchased from Chinese academy sciences (Shanghai, China). Briefly, PC12 cells were cultured in Dulbecco’s Modified Eagle’s Medium (DMEM) containing 10% fetal bovine serum (FBS; Hyclone), 0.37% NaHCO_3_, 100 U/mL penicillin, and 100 mg/mL streptomycin, in a humidified atmosphere with 5% CO_2_ at 37 °C. The medium was changed every 2 to 3 days. Cells were cultured to 70–80% confluence, and passaged twice a week.

Cell viability assay was performed according to MTT test. PC12 cells were plated in 96-well plates at a density of 5000 cells/well. When cells grew to 70–80% confluence, polysaccharides with different concentrations (0.125, 0.25, 0.5 and 1.0 mg/mL) were added to the cells and incubated for 1 h. Then the cells were treated with 50 μmol/L 6-OHDA, and cultured for 24 h, following the MTT solution at a dilution of 1/10 with 10% FBS DMEM was added to each well and the cells were incubated for 4 h at 37 °C. Absorbance at 570 nm was measured with a microplate reader (Biotek, MQX200). Cell viability= (A_blank_ − A_treatment_)/(A_blank_ − A_control_) × 100%. A_control_ was the absorbance of control 6-OHDA without the tested samples, A_treatment_ is the absorbance in the presence of the tested samples and A_blank_ is the absorbance with no samples or 6-OHDA but saline solution [20].

The LDH, SOD and MDA levels of PC12 were measured using commercial kits (Nanjing Jiancheng Bio, China), according to the manufacturer’s instructions. 

Statistical analysis: All data obtained in this study were processed statistically and divergences were presented as mean ± SD. SPSS 16.0 for Windows was used and *p <* 0.05 indicated significance differences.

## 4. Conclusions and Discussion

A heteropolysaccharide, SV2-1 with a molecular weight of 23 kDa was isolated from the viscera autolysates of squid (*Ommastrephes bartrami*) as the major polysaccharide component. The structure of SV2-1 was found to be a backbone consisting of alternate →4)-α-l-Fuc*p*-(1→ and →3)-β-d-GlcUA-(1→ at a molar ratio of 1:1, and with a single branch of α-*N*-acetylgalactosamine at the C-3 position of the 90% of the fucose according to chemical analysis and NMR analysis. 

It is interesting that many examples reveal marine animals contain abundant fucosylated polysaccharides. Those polysaccharides isolated from sea cucumber and cephalopod also have GlcA, GalNAc and Fuc as the monosaccharide composition could illustrate that fact [29]. However, the structures are different from SV2-1. The heteropolysaccahride from sea cucumber, which is called fucosylated chondroitin, has a repeating disaccharide unit of GlcA-GalNAc at the backbone with fucose as the branch, rather than Fuc at the backbone with GalNAc as the branch as the SV2-1. The polysaccharide isolated from the ink of cuttlefish *S. maindroni*, also has Fuc at the backbone, which is the same as SV2-1. By contrast, the polysaccharide isolated from *S. maindroni* was composed of Fuc and GalNAc at the backbone with GlcUA as the branch [6].

SV2-1 can protect PC12 cells from the death induced by 6-OHDA. Oxidative stress has been considered as a major factor caused by 6-OHDA. Oxygen tension regulates apoptosis in PC12 cells. SOD is a powerful antioxidant that catalyzes redox reactions by converting superoxide radicals into hydrogen peroxide and oxygen, which plays a beneficial role in the response of the brain to oxidative stress and protects brain tissues from further oxidative damage. Meanwhile, SOD reflects the level of ischemic tissue. MDA, a stable end product of lipid peroxidation, is responsible for ROS-mediated oxidative stress and implicated in disease severity [20,22]. In the present study, oxidative injury in PC12 was determined by measuring SOD activity and MDA concentration. SV2-1 significantly reversed the abnormorality of SOD and MDA. This result indicated that SV2-1 may normalize the redox status of PC12 cells under 6-OHDA injury and the underlying mechanism may be involved in its antioxidant capacity.

## Figures and Tables

**Table 1:** Gas chromatography-mass spectrometry (GC-MS) data analysis of partial *O*-methylated alditol acetates of SV2-1 and SV2-1P.

Methylation Product	Main MS (*m*/*z*)	Molar Ratio (%)		Linkage Pattern
		SV2-1	SV2-1P	
1,4,5-tri-*O*-acetyl-2,3,6-tri-*O*-methyl-l-fucitol	101,117,143,203	1	67	→4)Fuc(1→
1,3,4,5-tetra-*O*-acetyl-2,6-di-*O*-methyl-l-fucitol	113,117,129,173,275	6	15	→3,4)Fuc(1→
1,3,5-tri-*O*-acetyl-2,4,6-tri-*O*-methyl-d-gluctitol	101,117,129,161,233	-	8	→3)Glc(1→

**Table 2:** ^1^H and ^13^C NMR chemical shifts (δ) for the residues of SV2-1.

Residue	H1/C1	H2/C2	H3/C3	H4/C4	H5/C5	H6/C6		
A Fuc	5.19	3.84	3.95	4.03	4.33	1.19		
	99.2	68.4	75.1	82	66.5	15.9		
B GalNAc	5.08	4.03	3.80	3.87	3.95	3.80, 3.61	CH_3_	C=O
							1.96	174
	98.8	50.2	67.5	68.8	74.5	61.2	22.0	
C GlcUA	4.47	3.55	3.62	3.66	-/-	-/174		
	103.9	74	78	70.5				
